# Inappropriate Shock from Subcutaneous Implantable Cardioverter-Defibrillator Caused by Electrical Remodeling After Wolff-Parkinson-White Ablation

**DOI:** 10.7759/cureus.7766

**Published:** 2020-04-21

**Authors:** Oranus Mohammadi, Matthew S Glassy, Brian Cheung, Umair Shaikh, Nayereh Pezeshkian

**Affiliations:** 1 Internal Medicine, Aventura Hospital and Medical Center, Aventura, USA; 2 Cardiology, UC Davis Medical Center, Sacramento, USA; 3 Anesthesiology, Kendall Regional Medical Center, Miami, USA; 4 Cardiac Electrophysiology, UC Davis Medical Center, Sacramento, USA

**Keywords:** inappropriate shock, subcutaneous implantable cardioverter, hypertrophic cardiomyopathy, electrical remodeling, wolff-parkinson-white, t-wave oversensing

## Abstract

Hypertrophic cardiomyopathy (HCM) and Wolff-Parkinson-White syndrome have been associated with sudden cardiac death. A subcutaneous implantable cardioverter-defibrillator (S-ICD) is an effective device used to reduce the risk of sudden cardiac death in these patients. The most common cause of inappropriate shocks with S-ICD is T-wave oversensing. We present the case of a 19-year-old man with repeated shocks from his S-ICD. This case highlights some of the sensing issues related to the S-ICD that can result in inappropriate shocks. A vector change may have occurred after T-wave remodeling, post accessory pathway ablation, and loss of R-waves due to HCM scar progression, leading to this consequence.

## Introduction

Familial hypertrophic cardiomyopathy (HCM) and Wolff-Parkinson-White (WPW) syndrome have been associated with sudden cardiac death. Genetic analysis of patients with HCM and pre-excitation has identified specific mutations to be more common in this group and associated with a higher risk of sudden cardiac death. Mutations in protein kinase adenosine monophosphate (AMP)-activated non-catalytic subunit gamma 2 (PRKAG2) or lysosome-associated protein-2 (LAMP-2) were recognized in some patients with HCM and WPW. A subcutaneous implantable cardioverter-defibrillator (S-ICD) is an effective alternative to transvenous ICD for reducing the risk of sudden cardiac death in patients with HCM. The most common cause of inappropriate ICD shock in patients with S-ICD is T-wave oversensing (TWOS), responsible for 64% to 85% of the incidents [1-6]. Therefore, pre-implant screening of the surface electrocardiogram with three vectors in various positions is essential in all patients with S-ICD to assure good sensing and prevent inappropriate shocks. We present a case of a 19-year-old male with HCM and WPW who was shocked inappropriately by S-ICD during exercise due to significant vector change post-implant.

## Case presentation

**Figure 1:**
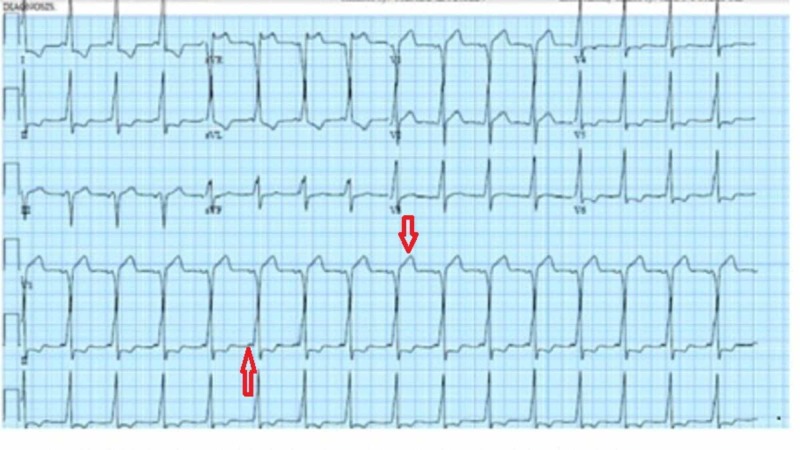
Post cardioversion electrocardiogram had revealed a short PR interval and pre-excitation

A 19-year-old Hispanic male presented to our emergency department with repeated shocks from his S-ICD. His past medical history was significant for ventricular fibrillation (VF) arrest while kneeling at church and having undergone cardiopulmonary resuscitation. Post cardioversion electrocardiogram (ECG) had revealed a short PR interval and pre-excitation (Figure 1).

**Figure 2:**
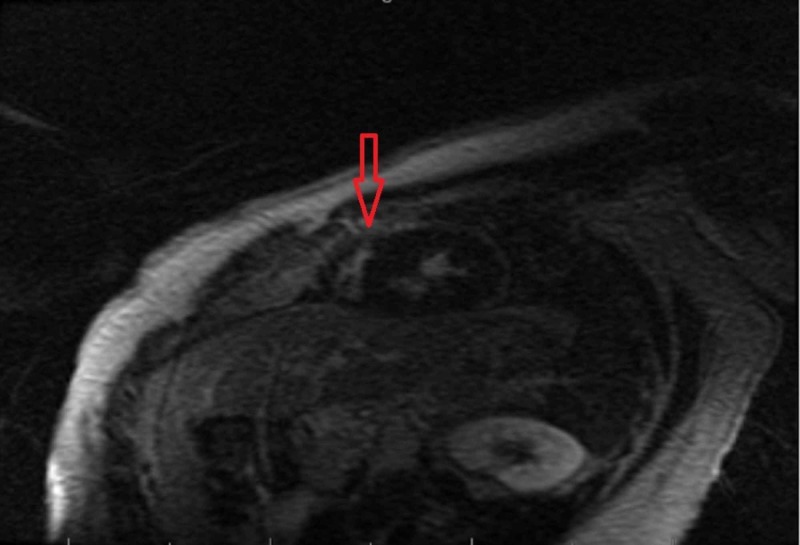
Cardiac magnetic resonance imaging showed thickening of the posterior septum with meso-myocardial delayed enhancement (red arrow)

Asymmetric concentric left ventricular hypertrophy with dynamic left ventricular outflow tract obstruction with a septum measured at 26 mm was noted on echocardiogram. Cardiac magnetic resonance imaging showed asymmetric thickening of the posterior septum with meso-myocardial delayed enhancement suggestive of HCM (Figure 2).

Subsequent genetic testing revealed myosin binding protein C3 (MYBPC3c.2149-1 G>A) heterozygosity. He then underwent a successful accessory pathway ablation. Since he had suffered VF arrest and was diagnosed with hypertrophic obstructive cardiomyopathy, an implantable defibrillator was considered. There was no pacing indication, and he passed screening ECG for S-ICD. Based on this, an S-ICD was implanted and tested. He then presented 21 months later with five S-ICD shocks. He reported doing jumping jacks in his bedroom for 20 seconds before the first shock. He then stopped, sat in a chair, and continued to receive four more shocks. He also reported he had been sedentary and had not taken his atenolol for one week prior to admission.

**Figure 3:**
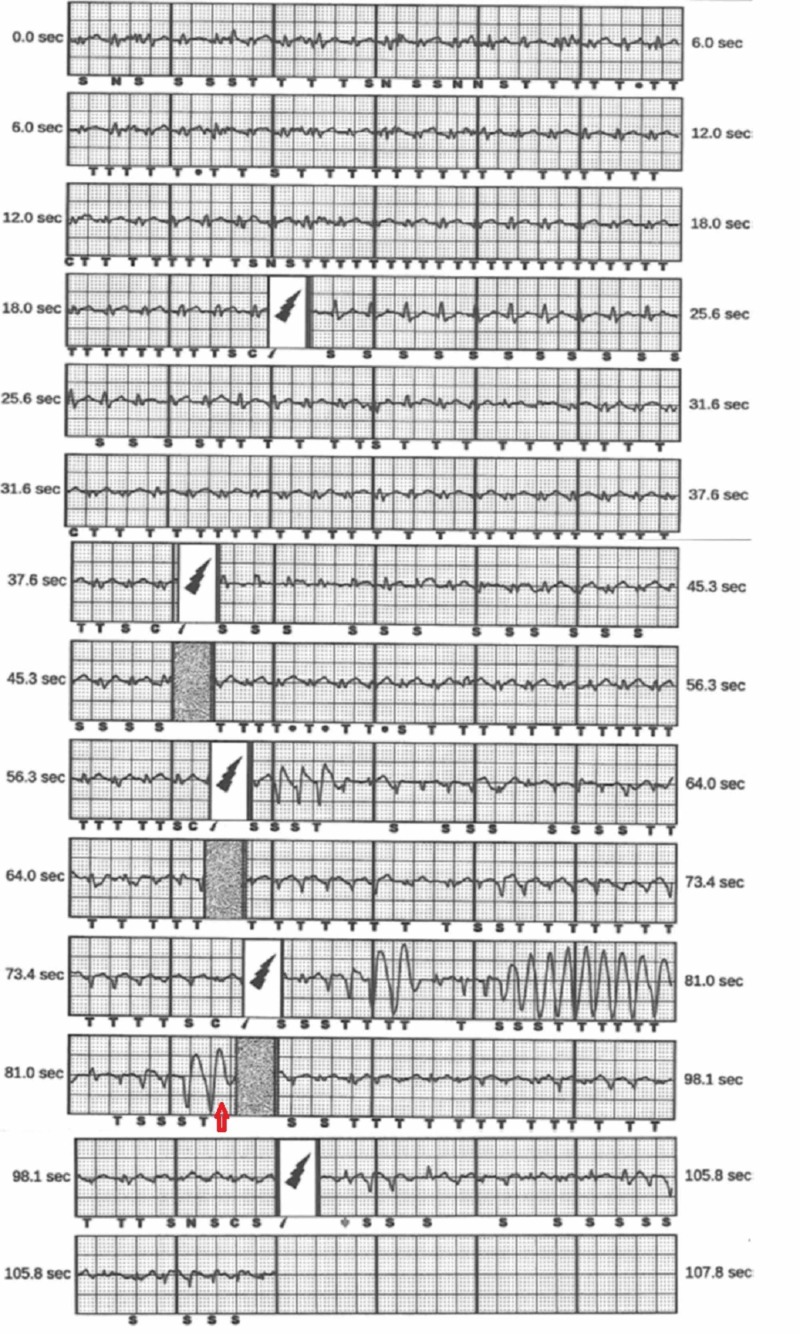
Interrogation of his subcutaneous implantable cardioverter defibrillator showed he was programmed to his secondary vector and revealed sinus tachycardia at 166 bpm prior to his shocks, starting at time point 0.

Interrogation of his S-ICD showed it was initially programmed using a secondary vector and revealed sinus tachycardia at 166 bpm prior to his shocks, starting at time point 0. Interrogation at the 30-second time point showed his QRS amplitude and QRS to T-wave ratio (QRS:T) became smaller after the 30-second time point, leading to the undersensing of the QRS and TWOS. This resulted in an inappropriate shock at 52 seconds. Then his QRS amplitude and QRS T briefly normalize for five seconds before decreasing again, and he received two additional inappropriate shocks due to TWOS. He developed non-sustained ventricular tachycardia after the third and fourth inappropriate shocks (Figure 3).

His potassium level was 3.8 mEq/L, bicarbonate level was 24 mEq/L, magnesium level was 2.0 mEq/L, and his toxicology screen was negative. His troponin I level was flat at 0.13 and 0.13, six hours apart. His chest x-ray demonstrated an appropriate S-ICD position.

We hypothesized that changes in the QRS axis noted in serial ECGs were the main reason for the change in QRS:T ratio and TWOS. These changes were likely caused by disease progression or T-wave remodeling over time after WPW ablation. Therefore, the secondary vector initially programmed was no longer sensing adequately. The jumping jack exercises, involving rhythmic arm movements, may have exaggerated the sensing problem while the patient was tachycardic. Also, we noted that the SMART low-pass filter (Boston Scientific Corporation, Natick, MA) was never activated in this device as the patient was lost to follow-up. SMART pass is a filter that reduces the T-wave’s amplitude without changing the frequency of R-waves and ventricular arrhythmias. He was rescreened and found to have much better sensing with the primary vector. The low pass filter was programmed for the primary sensing vector. The device was tested for sensing during treadmill and arm exercises. We performed a standard Bruce protocol exercise treadmill stress test with continuous interrogation of his primary vectors. He was able to reach Bruce stage 3 at six minutes and 27 seconds and achieve 7.6 metabolic equivalents (ml/kg/min) with a peak heart rate of 206 beats per minute (102% max predicted). Interrogation during this time and arm exercises showed no TWOS or QRS undersensing. His beta-blocker was reinitiated. At his 20-month follow-up evaluation, he has continued exercise with no mis-sensing.

## Discussion

This case highlights some of the sensing issues related to the S-ICD that can result in inappropriate ICD shocks. Changes in sensing over time due to the development of aberrancy and substrate progression after septal ablation in HCM have been described previously [7-9]. In our patient, a vector change may have occurred after T-wave remodeling, post accessory pathway ablation, and, more likely, loss of R-waves, probably due to HCM scar progression. Exercise-optimized reprogramming of the vector is one of the solutions to reduce the chance of inappropriate shocks [10]. We were able to program our patient’s device to the primary vector and add a SMART low-pass filter [11]. Subsequently, sensing was retested at rest, on standing, and during treadmill and jumping jack exercises. Follow-up ECG helped to understand vector change. Reprogramming should be considered before an attempted transvenous ICD implant.

## Conclusions

Several sensing issues in S-ICD can result in inappropriate shocks. In patients with underlying cardiac conditions like HCM or in those with a history of ablation, sensing issues in S-ICD use introduce the chance of vector change. This case shows vector change after T-wave remodeling, post accessory pathway ablation, and loss of R-wave likely due to disease progression.
